# Evolution of the Primate APOBEC3A Cytidine Deaminase Gene and Identification of Related Coding Regions

**DOI:** 10.1371/journal.pone.0030036

**Published:** 2012-01-17

**Authors:** Michel Henry, Christophe Terzian, Martine Peeters, Simon Wain-Hobson, Jean-Pierre Vartanian

**Affiliations:** 1 Molecular Retrovirology Unit, Institut Pasteur, Paris, France; 2 UMR754, UCBL-INRA and Ecole Pratique des Hautes Etudes, Lyon, France; 3 Institut de Recherche pour le Developpement (IRD) and University of Montpellier 1, UMI 233, Montpellier, France; Institut Pasteur Korea, Korea, Republic of Korea

## Abstract

The APOBEC3 gene cluster encodes six cytidine deaminases (A3A-C, A3DE, A3F-H) with single stranded DNA (ssDNA) substrate specificity. For the moment A3A is the only enzyme that can initiate catabolism of both mitochondrial and nuclear DNA. Human A3A expression is initiated from two different methionine codons M1 or M13, both of which are in adequate but sub-optimal Kozak environments. In the present study, we have analyzed the genetic diversity among *A3A* genes across a wide range of 12 primates including New World monkeys, Old World monkeys and Hominids. Sequence variation was observed in exons 1–4 in all primates with up to 31% overall amino acid variation. Importantly for 3 hominids codon M1 was mutated to a threonine codon or valine codon, while for 5/12 primates strong Kozak M1 or M13 codons were found. Positive selection was apparent along a few branches which differed compared to positive selection in the carboxy-terminal of A3G that clusters with A3A among human cytidine deaminases. In the course of analyses, two novel non-functional A3A-related fragments were identified on chromosome 4 and 8 kb upstream of the *A3* locus. This qualitative and quantitative variation among primate *A3A* genes suggest that subtle differences in function might ensue as more light is shed on this increasingly important enzyme.

## Introduction

The APOBEC3 seven gene cluster (A3A-C, A3DE, A3F-H) encodes six cytidine deaminases with single stranded DNA (ssDNA) substrate specificity [1], [2], [3], [4], [5], [6], [7], [8], [9]. Several are clearly innate restriction factors for viruses, notably for retroviruses, hepadnaviruses or parvoviruses [3], [5], [6], [10], [11], [12], [13], [14], [15], [16], [17], [18], [19], [20], [21], [22]. A3G and A3F constituted such a strong barrier for the lentiviral group of retroviruses that all but one encode a *vif* gene whose protein (Vif) is a powerful antagonist [3], [5], [6], [7], [19], [23], [24], [25], [26]. Hepatitis B virus (HBV) is restricted by at least two A3 enzymes while herpes simplex virus type 1 is restricted by A3C [20], [22], [27]. To date there are no reports of A3 antagonists encoded by these viral genomes. This antiviral role fits with the repeated observation that several A3 genes are up-regulated by type I and II interferons [10], [28], [29], [30], [31]. However, recent work has shown that this antiviral role is just part of a bigger picture [30], [32], [33]. For example, A3A can restrict Line transposition [11], [34], [35], [36]. Several A3 enzymes can initiate catabolism of mitochondrial DNA, in which uracil N-glycosylase plays a major role downstream of editing [33]. For the moment A3A is the only enzyme that can initiate catabolism of both mitochondrial and nuclear DNA [33].

These A3 proteins mediate hydrolytic deamination at the C4 position that oxidises cytosine to uracil in ssDNA so generating C→U hyper-edited molecules [1], [3], [5], [6], [7], [9], [22], [37], [38]. The active sites of A3 enzymes are characterized by a conserved zinc-finger HAEX_23–28_PCX_2–4_C motif [4]. These A3 enzymes show a strong preference for cytidine deamination occurring segment carboxy-terminal to the zinc finger impacts this dinucleotide specificity [39]. Human A3A expression is initiated from two different methionine codons (M1 and M13), both of which are in adequate but sub-optimal Kozak environments [40].

Even though a number of primate genomes are available, only the chimpanzee locus is colinear. For the orang-utan the *A3A* gene is incomplete while the entire locus contains 12 exon 3/exon 6 domains rather than 11. The *A3A* gene is missing in the Rhesus macaque assembly, while the marmoset locus doesn't exist *per se*, sequences being distributed over numerous contigs. As the *A3* locus shows signs of extensive gene conversion, the apparent gaps might reflect assembly problems.

We have analyzed the genetic diversity among *A3A* genes across a wide range of primates including New World monkeys, Old World monkeys and Hominids. There is variation among the Kozak motifs with the M1 initiator methionine being absent for chimpanzees, bonobos and gorillas. Some, but not all, *A3A* lineages show positive selection suggesting that A3A enzymes may not be truly orthologous.

## Results

### Primate A3A cytidine deaminases

**Figure 1:**
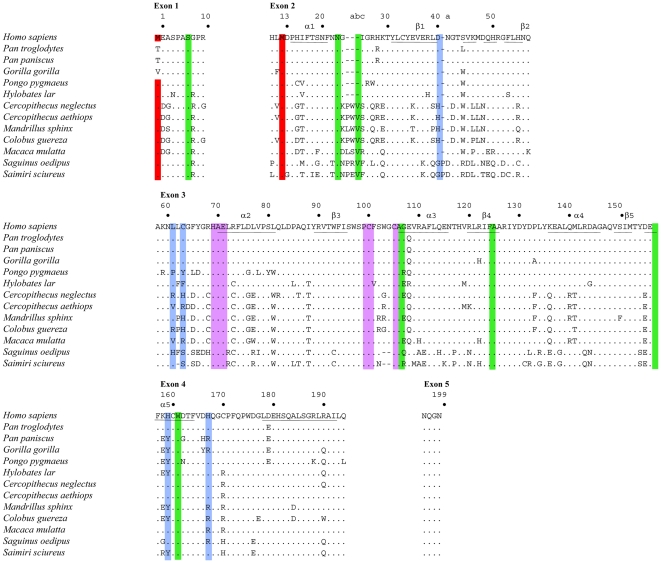
Alignment of primate APOBEC3A proteins. Twelve primate sequences were compared to *Homo sapiens* used as reference. Only differences are shown. Hyphens denote gaps introduced to maximize sequence identity. The numbering corresponds to that of the human sequence. The letters a, b, c are added to adjacent residue to accommodate insertions. Red denotes the first (M1, exon 1) and second initiation start codons (M13, exon 2). The crucial cytidine deaminase motif residues are highlighted in magenta. Positively and negatively selected codon sites are in blue and green respectively. The predicted secondary structure motifs for hA3A are underlined.

Twelve primates A3A sequences spanning New and Old World monkeys were derived by amplification of genomic DNA and given aligned to the human sequence (Figure 1). The A3A protein is initiated at codons M1 or M13 giving rise to two different proteins both with ssDNA cytidine deaminase activity [40]. The Kozak context of both human A3A initiator codons is considered to be adequate. For 3 hominids, codon M1 was mutated to a threonine codon or valine codon which probably abrogates translation initiation (Figure 1, Table 1). For both New World monkey sequences, the M1 Kozak context was strong suggesting that translation initiation at M13 would be reduced. In addition, the Kozak context of the M13 codon was strong for 3/12 primates notably *C. guereza*, *C. aethiops* and *C. neglectus* (Table 1). For all the others, the context is considered to be adequate for translation initiation.

**Table 1:** Kozak sequence contexts surrounding the A3A M1 and M13 initiation codons.

Monkey	Met1	Kozak context	Met13	Kozak context
*H. sapiens*	CACATGG	A (dequate)	TTGATGG	A
*P. troglodytes*	CACACGG	N(ull)	TTGATGG	A
*P. paniscus*	CACACGG	N	TTGATGG	A
*G. gorilla*	CACGTGG	N	TTCATGG	A
*P. pygmaeus*	CACATGG	A	TTGATGG	A
*Hylobates lar*	CACATGG	A	TTGATGG	A
*C. neglectus*	CACATGG	A	GTGATGG	S(trong)
*C. aethiops*	CACATGG	A	GTGATGG	S
*C. guereza*	CACATGG	A	GTGATGG	S
*M. sphinx*	CACATGG	A	TTGATGG	A
*M. mulatta*	CACATGG	A	TTGATGG	A
*S. oedipus*	GACATGG	S	CTGATGG	A
*S. sciureus*	GACATGG	S	CTGATGG	A

Sequence variation was observed in all exons apart from the very small exon 5. Some of the exon 5 sequences differ compared to some recently reported [35]. On a pairwise basis up to 31% amino acid divergence was observed overall, with 6%, 21% and 30% among hominids, Old World small monkeys and New World monkeys respectively. That the variation is as great overall as that between the New World monkeys, suggests that there has not been too much gene conversion in the New World lineage. Exon 3 encodes the hallmark HXEX_23–28_PCX_2–4_C motif for cytidine deaminases (Figure 1). Among all the human A3 enzymes only A3A encodes the PCX_4_C variant. Interestingly, the New World A3A sequences are singular in that they encode the PCX_2_C variant typical of all other A3 enzymes.

### A3A is under positive selection in Old World monkeys

In order to characterise whether this variation shows signs of selection, we estimated the relative numbers of non-synonymous (dN) and synonymous (dS) nucleotide substitutions per site and dN/dS ratios over the twelve primate species using the Hyphy package and FEL and REL methods [41]. We investigated models in which the dN/dS ratio is allowed to vary among the complete sequence using the GA-branch analysis. There was significant positive selection with estimated dN/dS ratios >1.0 (p>0.95), at five sites, notably D41, L62, C64, H160 & H168 (Figure 1, in blue). By contrast, several sites were under significant negative selection, notably S7, N24, V25c, A107, F125, E157 and W162 (Figure 1, in green).

**Figure 2:**
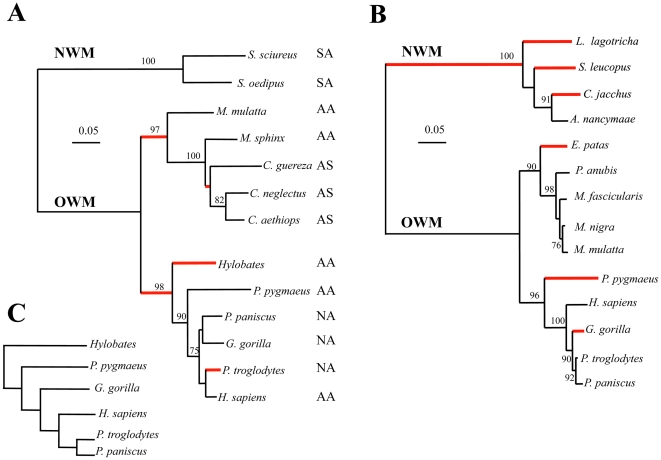
Phylogeny of primate A3A and A3Gc cytidine deaminases. A) Neighbor-joining tree based on A3A primate sequences presented in Figure 1. Only bootstrap values >70 are indicated. The nomenclature for the Kozak initiation sites (A: adequate, N: null, S: strong) are from Table 1. B) Neighbor-joining tree based on A3Gc primate sequences taken in the literature. For both, red branches correspond to class d_N_/d_S_>1 (p>0.9) while the remainder correspond to classes d_N_/d_S_<1. C) The accepted divergence of the Great Apes.

A phylogenic tree for the complete sequence of A3A was constructed using BioNJ (Figure 2A). The red internal branches denote those where dN/dS>1 (p>0.9) which are confined to a small fraction of the total number of branches. Among A3 enzymes, the A3A sequence is phylogenically closest to the carboxy-terminal domains of A3B and A3G. In view of a large collection of A3G sequences [42], a comparable analysis was made using the A3Gc sequences (Figure 2B). The branch-specific patterns of dN/dS variation for both *A3A* and *A3Gc* cytidine deaminases are different, a good example being the New World monkey lineage.

### An A3A exon 3-related sequence on human chromosome 4

**Figure 3:**
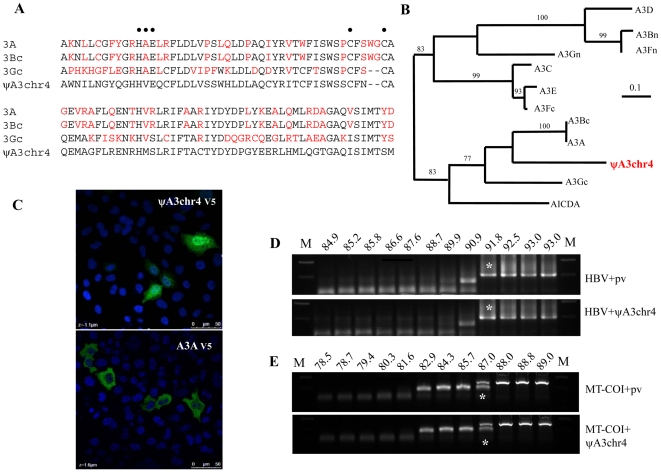
No cytidine deaminase activity associated with pΨA3chr4. A) Alignment of exon 3 of ΨA3chr4 proteins to human A3Bc, A3Gc and A3A. Differences with respect to ΨA3chr4 are highlighted in red. B) Neighbor-joining tree based on the exon 3 of human *A3* genes. Only bootstrap values of >70 are given. C) Immunostaining of the V5-tagged pΨA3chr4 and A3A proteins with DAPI nuclear counterstain. D) Agarose gel of 3DPCR products across a denaturation temperature gradient from 93 to 85°C of X region of HBV. pCayw is an infectious molecular clone along with the empty expression vector, along with pΨA3chr4. M; molecular weight markers. The white asterisks denote the last amplification product obtained at 91.8°C. E) Agarose gel of 3DPCR products across a denaturation temperature gradient from 89 to 79°C on *MT-COI* gene. The white asterisks denote the last amplification product of *MT-COI* obtained at 87°C.

When performing Blat searches for this study (UCSC Genome Bioinformatics), we identified a segment of 288 bp on human chromosome 4 with strong homology to exon 3 of the A3A/A3Bc/A3Gc cluster (Figure 3A) which will be referred to as ΨA3chr4. Homology went out to a few hundred bases either side with the splice sites perfectly conserved. The sequence is present in human, chimpanzee, gorilla, orang-utan, macaque and marmoset genomes while absent in horse, dog, cat and rodent genomes. At the protein level, the exon revealed a HVEX_n_SCX_2_C motif similar to that for all A3 deaminases (HAEX_n_PCX_2–4_C) (Figure 3A). While the A→V substitution is found in AID and APOBEC1 sequences, the P→S substitution is without precedent. Phylogenic analysis based on amino acid sequences showed that it emerged after the (A3A, A3Bc)A3Gc split (Figure 3B).

5′ and 3′ RACE failed to identify any transcripts while no EST was found in the databases. Nonetheless, to ascertain whether this exon encoded a functional domain, we synthesized a fusion gene with the exon surrounded by exons 1, 2, 4 and 5 of the human *A3A* gene. The construct was cloned in pcDNA3.1 TOPO resulting in addition of the V5 tag. When transfected into HeLa cells and stained with FITC-conjugated anti-V5 antibody the construct was viable and strongly nuclear, more so than hA3A indicating that residues impacting A3A localization lie in exon 3 (Figure 3C). In order to demonstrate editing activity, HeLa cells were co-transfected by the reconstructed pΨA3chr4 clone and an infectious molecular clone of hepatitis B virus. Total DNA was analysed at 72 hrs by a nested PCR/3DPCR approach as previously described [22], [43].

The minimal denaturation temperature (Td) for the HBV X gene segment analysed is 91.8°C (Figure 3D, [22]). When co-transfected with the reconstructed pΨA3chr4 clone, the lowest Td was equally 91.8°C indicating that the recombinant may not be packaged into assembling HBV virions. Accordingly, a non-viral region corresponding to *MT-COI* gene was been amplified by PCR/3DPCR [33]. The minimal Td for *MT-COI* DNA was 87°C with or without pΨA3chr4 (Figure 3E), suggesting that the chromosome 4 fragment is indeed devoid of ssDNA cytidine deaminase activity.

Finally, an additional ∼1.1 kb *A3A*-related fragment was identified ∼8 kb upstream of the human *A3A* gene in the same orientation as the entire *A3* locus. For comparison, the *A3A-A3B* intergenic region is ∼19 kb. The fragment comprises 104 bp (37%) of intron 4, exon 5 and downstream sequences. Overall it shows 96% nucleic acid homology to *hA3A*. As such it must represent a vestige of prior gene conversion. Indeed, the sequence is surrounded by repeat elements, some of which are found surrounding the *hA3A* and *hA3B* genes. This *A3A* remnant is found in the chimpanzee, orang-utan and rhesus macaque genomes.

## Discussion

The primate *A3A* gene shows considerable qualitative and quantitative genetic variation, with up to 31% amino acid variation. Translation initiation sites vary there being at least four different configurations (Table 1). Positive selection is apparent along a few but not all branches suggesting that differences may emerge when more attention is turned to this important enzyme.

From the outset, differences in the restriction patterns of primate A3G on HIV-1Δ*vif* were noted [44], [45], [46]. More recent reports show that several human and macaque A3 cytidine deaminases are not strictly equivalent when using HIV-1 as a readout [47]. Indeed, as several reports have shown subtle differences for A3B, A3DE and A3G [47], [48], [49], this should transpire for A3A. However, as this enzyme impacts the integrity of the human genome, it is possible that the variation in structure and evolution of the *A3A* gene could impact cell biology.

During data analyses, two *A3A* related fragments were identified. The ΨA3chr4 exon 3 fragment proved to be devoid of catalytic activity when spliced together with exons 1, 2, 4 and 5 from *A3A*. This solo A3 exon is reminiscent of the recent finding of an isolated APOBEC1 exon in the tetrapod lineage that was subsequently lost [50]. The second *A3A* fragment is particularly interesting in that it shows that the present organization of the primate *A3* locus might well have come about via more gene conversion than previously thought [51]. In conclusion, there is subtle qualitative and quantitative variation among primate *A3A* genes. In turn, gene expression and perhaps interferon sensitivity might follow.

## Materials and Methods

### Animal samples

Faecal samples were collected from wild non-habituated western gorilla (*Gorilla gorilla gorilla*) and chimpanzees (*Pan troglodytes troglodytes*) in Cameroun with permission of the Cameroonian Ministries of Health, Research and Environment and Forestry and Wildlife, and from bonobo (*Pan paniscus*) in the Democratic Republic of Congo with the permission of the Ministries of Science and Technology and Forest Economy [52]. DNA was extracted as previously described [53]. For mantled guereza (*Colobus guereza*) and mandrills (*Mandrillus sphinx*), DNA was extracted from whole blood on samples that were collected on primate bushmeat with permission from Cameroonian Ministries of Health, Research and Environment and Forestry and Wildlife, as previously reported [54]. Primary cells and cells line were obtained for orang-utan (*Pongo pygmaeus*) that died of natural causes while housed at the Wanariset orang-utan Reintroduction Center in East Kalimantan, Indonesia [55] and white-handed gibbon (*Hylobates lar*, ATCC 57763) respectively, while samples of rhesus monkey (*Macaca mulatta*, ATCC CCL-7), vervet monkey (*Cercopithecus aethiops*, ATCC CCL-81), and necropsy tissue samples from a squirrel monkey (*Saimiri sciureus*) and cotton-top tamarin (*Sanguinus Oedipus*) that died of natural causes while kept in a zoo have been already described [56]. Primary cells from De Brazza's monkey (*Cercopithecus neglectus*) came from an animal that died of natural causes while housed at the zoo de la Palmyre (France).

### PCR amplification, cloning and sequencing

Hot start PCR was performed with corresponding primers (Table 2). The first reaction involved standard amplification, the reaction parameters were 95°C for 5 min., followed by 35 cycles (95°C for 30 s., 50–55°C for 30 s. and 72°C for 1 min.) and finally for 10 min. at 72°C for the first round. Differential amplification occurred in the second round using the equivalent of 0.2 µL of the first round reaction as input. Conditions were identical to the first PCR. The buffer conditions for all amplification were 2.5 mM MgCl_2_, 50 mM KCl, 10 mM Tris-HCl pH 8.3, 200 µM of each dNTP, 100 µM of each primer, and 2.5 units of BIOTaq polymerase (Bioline) in a final volume of 50 µL. PCR products were purified from agarose gels (Qiaex II kit, Qiagen, France) and ligated into the TOPO TA cloning vector (Invitrogen, France). After transformation of Top10 electrocompetent cells (Invitrogen), up to 15 clones were picked. Sequencing was outsourced to GATC biotech. All mutations were confirmed by inspection of the chromatogram. The pΨA3chr4 insert was synthetized by GeneCust and cloned into the pcDNA3.1 TOPO-V5 vector (Invitrogen).

**Table 2:** Compendium of all PCR primers used in the study.

Primer	Sequence
E1out5	5′ AGAACTTCCTTGTTCTGATGCTAATGTGGGTGG
E1out3	5′AAAGGGAGGCCCCGGGGTTTGAGGGTGA
E1int5	5′ GGCCTGAGCTGGAGAAGGGGTGGGGCAG
E1int3	5′ TCAGAAAAGCTCAAAGAGGAGGCTGAGG
E2out5	5′ GGCCTGAGCACACTGAGCTGACCCTGGG
E2out3	5′ GAATGTCCCTGGAYTGGRAGGGCCCTG
E2int5	5′ GAGACCCTGACAAGGCTTAGACA
E3out5	5′ TGGTCCAGGCCCTCCCTCCCTGTTCAC
E3out3	5′ CARGCARCAGMCCAAGGCCTTTCTCC
E3int5	5′ ACCTCACACTCTGTTTCCTTTTCTA
E3int3	5′ CCCACCCCACCCCACCTGAACCTTCC
E4out5	5′ CGGGARYGKGACTTATCTCYCCTGTC
E4out3	5′ GGAGGWGGCCTGGGGACAGKGACAATGA
E4int3	5′ GGGAGAAGGAGGGAGGGATGCGGGAGG
E5out5	5′ TGTGCCCTCTTTCCACTCTCTCACCTCC
E5out3	5′ TCTGCTGCTCAACCCAGGTCTCT
E5int5	5′ CCACTCTCTCACCTCCTGCTCCA
E5int3	5′ TCTCTGCCTTCCTTAGAGACTGA

### Cells and transfections

Briefly, 10^5^ QT6 cells (ATCC CRL 1708) were cotransfected with 1 µg of pΨA3chr4 plasmid DNA along with 1 µg pCayw, a plasmid encoding an infectious molecular genome of hepatitis B virus (HBV) using FuGENE 6 (Roche). Total DNA was extracted using the MasterPureTM complete DNA and RNA purification kit (Epicentre). QT6 cells were maintained in HAM's F40 medium, supplemented with 1% chicken serum, 10% FCS, 5% tryptose phosphate, 2 mM L-glutamine, 50 U/ml penicillin and 50 µg/ml streptomycin [15].

### Immunofluorescence

HeLa cells (ATCC CCL 2) were grown to a density of 5.10^5^ cells per dish [15] and transfected with 1 µg of pΨA3chr4 or pA3A using FuGENE 6 (Roche). After 48 hours, the cells were washed twice with PBS, fixed for 45 minutes in a 50∶50 methanol/ethanol mix. As primary antibodies, a mouse monoclonal antibody specific for the V5 epitope tag (Invitrogen) was used at a 1∶200 dilution for 1 hour at room temperature. Cells were washed twice with PBS, and FITC-conjugated anti-mouse antibody anti-mouse was used as second antibody (Sigma) at a dilution 1∶200 for 30 minutes at room temperature. We used Vectashield, mounting medium for fluorescence with DAPI (Vector laboratories, Inc.). Immunofluorescence was observed by microscopy (Zeiss).

### Phylogenic and computational analyses

Sequences were aligned using the MUSCLE program, and neighbor-joining trees were obtained using BioNJ as implemented in http://phylogeny.fr. The final output was edited using Treeview [57]. The relative numbers of non-synonymous (dN) and synonymous (dS) nucleotide substitutions per site were estimated using the random effects likelihood (REL) and the fixed effects likelihood (FEL) methods available via the Datamonkey web interface of the HyPhy package [58]. Estimates of dN/dS ratios were based on neighbor-joining trees obtained from phylogeny.fr.

We used the genetic algorithm (GA-Branch) method available in HyPhy [58] to detect lineage-specific variation in selection pressure. This assigns different classes of dN/dS ratios to each lineage to determine the best-fit model of lineage-specific evolution, and it calculates the probability (≥90%) that along a specific lineage dN/dS>1 [41].

Accession numbers were deposited at GenBank: *Colobus guereza* (JN177339), *Cercopithecus aethiops* (JN177340), *Cercopithecus neglectus* (JN177341), *Mandrillus sphinx* (JN177342), *Macaca mulatta* (JN177343), *Hylobates lar* (JN177344), *Gorilla gorilla* (JN177345), *Pongo pygmaeus* (JN177346), *Pan paniscus* (JN177347), *Pan troglodytes troglodytes* (JN177348), *Saguinus oedipus* (JN177349) and *Saimiri sciureus* (JN177350).
